# Lung Epithelial Injury by *B. Anthracis* Lethal Toxin Is Caused by MKK-Dependent Loss of Cytoskeletal Integrity

**DOI:** 10.1371/journal.pone.0004755

**Published:** 2009-03-09

**Authors:** Mandy Lehmann, Deborah Noack, Malcolm Wood, Marta Perego, Ulla G. Knaus

**Affiliations:** 1 Department of Immunology & Microbial Science, The Scripps Research Institute, La Jolla, California, United States of America; 2 Core Microscopy Facility, The Scripps Research Institute, La Jolla, California, United States of America; 3 Department of Molecular & Experimental Medicine, The Scripps Research Institute, La Jolla, California, United States of America; LMU University of Munich, Germany

## Abstract

*Bacillus anthracis* lethal toxin (LT) is a key virulence factor of anthrax and contributes significantly to the *in vivo* pathology. The enzymatically active component is a Zn^2+^-dependent metalloprotease that cleaves most isoforms of mitogen-activated protein kinase kinases (MKKs). Using *ex vivo* differentiated human lung epithelium we report that LT destroys lung epithelial barrier function and wound healing responses by immobilizing the actin and microtubule network. Long-term exposure to the toxin generated a unique cellular phenotype characterized by increased actin filament assembly, microtubule stabilization, and changes in junction complexes and focal adhesions. LT-exposed cells displayed randomly oriented, highly dynamic protrusions, polarization defects and impaired cell migration. Reconstitution of MAPK pathways revealed that this LT-induced phenotype was primarily dependent on the coordinated loss of MKK1 and MKK2 signaling. Thus, MKKs control fundamental aspects of cytoskeletal dynamics and cell motility. Even though LT disabled repair mechanisms, agents such as keratinocyte growth factor or dexamethasone improved epithelial barrier integrity by reducing cell death. These results suggest that co-administration of anti-cytotoxic drugs may be of benefit when treating inhalational anthrax.

## Introduction

Inhalational anthrax is an infectious disease caused by the uptake of aerosolized spores of the gram-positive bacterium *Bacillus anthracis* into the airways. Alveolar macrophages and lung-resident dendritic cells ingest these spores and migrate to nearby lymph nodes, where intracellular germination and release of vegetative bacteria occurs. After a slow onset *B. anthracis* divides rapidly in the circulatory system, resulting in fulminant disease characterized by respiratory distress, haemorrhage, shock and sudden death of the host [1], [2]. The rapid and lethal development of *B. anthracis* sepsis is triggered by massive release of a tripartite bacterial exotoxin [3], [4]. Anthrax toxin consists of protective antigen (PA), lethal factor (LF) and edema factor (EF). PA constitutes the receptor-binding and carrier protein, which is responsible for host cell attachment, complex formation with LF and EF, cellular entry of the toxin complex and intracellular release of LF and EF to endocytic organelles or the cytoplasm [5]. EF is a calcium- and calmodulin-dependent adenylate cyclase that raises intracellular levels of cAMP, a second messenger altering the activity of signaling molecules including guanine nucleotide exchange factors (i.e. Epac), protein kinases (i.e. PKA) and certain ion channels [6]. The pathological consequences of the combined binary edema toxin (ET; PA and EF) range from imbalance of fluid homeostasis and edema formation in lungs to inhibition of chemotaxis and phagocytosis, and suppression of angiogenesis in endothelial cells. LF is a Zn^2+^-dependent metalloprotease that specifically cleaves almost all of the mitogen-activated protein kinase (MAPK) kinases (MKKs) [7], [8]. The cleavage occurs close to the N terminus of MKKs and removes the so-called D-domain, a MAPK docking motif, thereby decreasing MKK-MAPK binding affinity and MKK-induced phosphorylation and activation of MAPK. MKKs provide a crucial link from many cell surface receptors to Erk, JNK and p38, and thus mediate the regulation of many transcriptional networks by mitogenic and stress signals. Binary lethal toxin (LT; PA and LF) has been connected to caspase-1-dependent cell death of susceptible innate immune cells, to endothelial cell apoptosis, and to cell cycle arrest in epithelial cells [9]. LT alters the host immune response by abolishing cytokine production and expression of co-stimulatory molecules. Recently, LT was linked to decreased neutrophil chemotaxis due to reduced actin filament assembly and to inhibition of actin-based motility of the intracellular pathogen *Listeria monocytogenes* in HeLa cells [10], [11]. Not all of LT's detrimental effects on host cells seem to be a consequence of abolished MKK function. Specifically, LT-induced cell death has not been conclusively linked to MKKs [3], suggesting that other LF target proteins may exist.

Although interference with anti-bacterial responses of the host innate and adaptive immune system is important for *B. anthracis'* ability to replicate rapidly, non-immune cells seem to contribute to the severity and fatality of *B. anthracis* infection when bacterimia-induced toxin concentrations reach considerable levels. Injection of mice with purified LT results in increased vascular leakage, pleural edema and hypoxic tissue injury [12]. Similarly, pathological features in systemic anthrax infection in Rhesus monkeys, chimpanzees and inhalational anthrax victims are hemorrhages, pleural effusion and edema [13]. This phenotype suggests that the barrier function of the endothelium might be affected by LT. In fact, LT caused significant endothelial barrier dysfunction in primary human microvascular endothelial cells [14]. This effect was not caused by apoptosis, although caspase-dependent apoptosis in LT-treated HUVEC was reported elsewhere [15]. The observed increase in permeability, causing fluid leakage, could not be mimicked by suppressing all three MAP kinase pathways with chemical inhibitors and thus, the underlying mechanism of endothelial junction disruption by LT remains unresolved.

The clinical manifestation of anthrax rarely includes bronchopneumonia in the earlier stages of the disease. In contrast, the final sepsis and shock stage of infection is characterized by respiratory distress and pulmonary edema. This may indicate that the airway epithelium plays not only a role in dissemination of *B. anthracis* infection [16], but that breakdown of the lung epithelial barrier is also a crucial, late event in anthrax lethality. In this study, we report that lethal toxin has a profound destructive effect on the barrier function of differentiated human airway epithelium. This LT-induced phenotype is the consequence of altering major components of the mammalian cytoskeleton and microtubule network, causing a state of permanent paralysis where turnover of structural components is greatly reduced. Lung epithelial cell functions, which require sustained dynamic regulation such as directed migration and multicellular junction reorganization, were abolished by LT. The structural paralysis of airway cells is MAPK pathway-dependent and can be efficiently counteracted by introducing non-cleavable MKK mutants. As this approach is currently not a therapeutic option, several compounds recognized for lung-protective function were assessed for their ability to reduce toxin-induced barrier damage.

## Results

### Lethal toxin destroys the barrier function of human mucociliary lung epithelium

**Figure 1:**
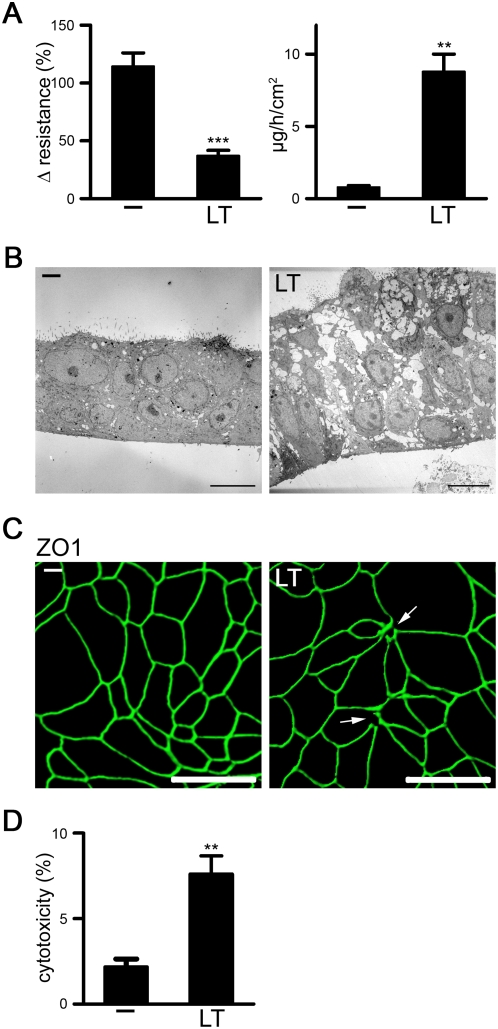
LT destroys barrier function in differentiated mucociliary human lung epithelium. (A–D) Polarized NHBE were treated with or without LT for 48 h. (A) The epithelial layer was subjected to resistance measurements (left panel) or incubated with FITC-albumin on the apical side for permeability measurements (right panel). Data are represented as mean+/−SEM of 3–5 inserts/per condition, n = 3. (B) TEM images of crosscuts obtained from untreated (-) and LT-treated lung epithelial layers. Crosscuts with ciliated cells on the apical side are depicted. Scale bar represents 10 µm. (C) Confocal images of the apical side of untreated and LT-treated lung epithelial layers using a tight junction marker (ZO1, green). Z-series was analyzed with Imaris5 (Bitplane Inc., MN). Disruptions of multicellular junctions are indicated by white arrows. The scale bar represents 50 µm. (D) LDH release of untreated and LT-treated NHBE 3D layers. Data are represented as mean+/−SEM of 3 inserts, n = 3.

To study the effect of *B. anthracis* toxins on the respiratory epithelium, a three-dimensional (3D) model of polarized, mucociliary lung epithelium was established. Primary human lung epithelial cells were grown on extracellular matrix-coated filters in air-liquid interface for 3–4 weeks. The differentiated phenotype was characterized by a marked morphological change, giving rise to a multicellular layer consisting of basal cells, non-ciliated cells, ciliated cells and goblet cells (Figure S1A). The *ex vivo* differentiated airway cells formed tight and adherens junction complexes, produced mucus and expressed defensins and surfactant (Figure S1B–D). Human lung epithelial cells expressed both anthrax receptors, TEM8/ATR and CMG2 (Figure S1E) as previously demonstrated in human lung tissue for TEM8 [17]. This model system was used to determine if circulating *B. anthracis* exotoxins alter lung epithelial cell functions. Addition of protective antigen (PA) alone or edema toxin (ET; PA and EF) into the media in the bottom chamber did not alter barrier function (Figure S2A), while basolateral application of lethal toxin (LT; PA and LF) caused a significant decrease in transepithelial electrical resistance and a substantial increase in permeability of the layer to fluorescently labeled albumin applied from the apical side (Figure 1A). Transmission electron microscopy revealed that treatment of the 3D epithelium with LT caused an expansion of the epithelial layer and the appearance of large paracellular spaces (Figure 1B). Staining of the untreated and LT-treated epithelium with the tight junction marker ZO1 showed disruption of the apical junction network at multicellular connection sites (Figure 1C). When exposed to LT, human lung epithelial cells cultured in typical 2D conditions, did not exhibit any signs of apoptosis as judged with caspase 3-mediated cleavage of poly (ADP-ribose) polymerase (PARP) cleavage, by analysis of phosphatidylserine exposure, by DNA laddering or by transferase mediated dUTP nick end labeling (TUNEL) staining. However, up to 5% of cells detached during the first 48 hours of treatment. This loss of attached cells did not accelerate substantially after prolonged incubation in growth factor-containing media, although LT-treated cells failed to resume proliferation (Figure S2B). In polarized lung epithelium a minor, but reproducible increase of cell death was observed after 48 hours of LT treatment (Figure 1D), which would be sufficient for loss of barrier function if junction complex remodeling is disturbed.

### Increase and redistribution of junction proteins by lethal toxin

**Figure 2:**
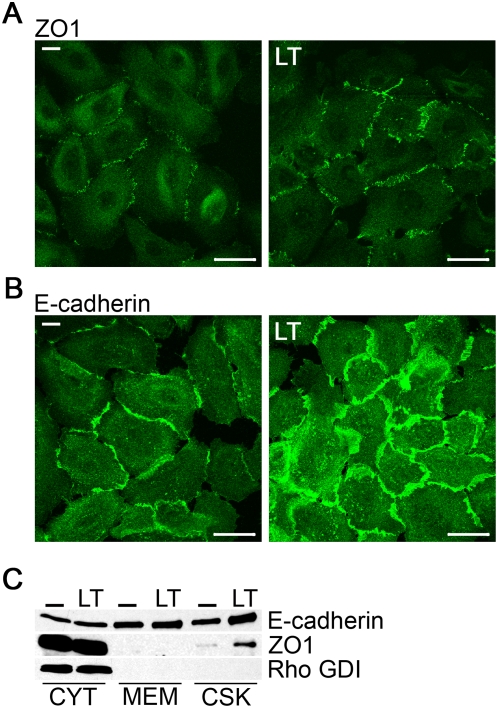
Junction proteins are elevated after LT treatment. (A, B) NHBE cells were treated with or without LT for 48 h and stained with ZO1 (A) or E-cadherin (B). Confocal images were taken consecutively. Scale bar represents 50 µm. (C) Fractionation of LT-treated and untreated (-) NHBE cells into cytosol (CYT), membrane (MEM) and cytoskeletal (CSK) fractions. Immunoblotting was performed using anti-E-cadherin and anti-ZO1. Rho GDI served as cytosol marker.

Comparison of tight junctions (TJ) in untreated versus LT-treated 3D model systems did not reveal clear differences in ZO1 distribution between two adjacent cells (Figure 1C). Analysis of lung epithelial cells grown in 2D conditions undergoing LT treatment clearly showed an increase of the TJ marker ZO1 along the border of adjoining cells (Figure 2A). ZO1 was concentrated in punctate structures lining cell-cell contacts. Similarly, the adherens junction (AJ) protein E-cadherin was up-regulated in cells exposed to LT (Figure 2B). Although the overall cell morphology of lung epithelial cells was retained, E-cadherin-containing cell junctions were less focused and more dispersed. Subcellular fractionation did not only confirm the confocal analysis, but indicated a redistribution and enrichment of both junction proteins in the cytoskeletal fraction (Figure 2C).

### Lethal toxin-induced reorganization of the cytoskeleton and microtubule network

**Figure 3:**
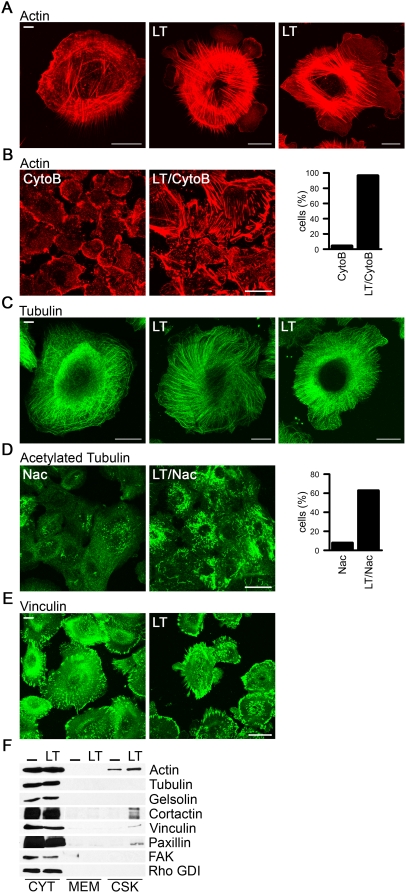
LT induces stress fiber formation and stabilized microtubules. (A, C) Immunofluorescence images of NHBE cells with or without LT treatment (48 h). Cells were stained for actin (A, red) or α-tubulin (C, green). Scale bar represents 20 µm. (B, D) Cells with or without LT treatment were incubated for 1 h with 1 µg/ml cytochalasin B (CytoB) (B) or 10 nM nacodazole (Nac) (D) and stained for actin (red, B) or acetylated tubulin (D, green). Scale bar represents 50 µm. Remaining actin stress fibers (B) or stable microtubules (D) were quantified in 500 cells/per condition. (E) Confocal images of NHBE cells treated with or without LT and stained with vinculin (green). Scale bar represents 50 µm. (F) Fractionation of LT-treated and untreated (-) NHBE cells into cytosol (CYT), membrane (MEM) and cytoskeletal (CSK) fractions. Immunoblotting was performed using the indicated antibodies. Rho GDI served as cytosol marker.

The apical junctional complex, composed of TJs and AJs, is intimately connected with the actomyosin cytoskeleton via several connections including a ZO1-cortactin linkage and an E-cadherin-catenin-Rho GTPase axis. Confocal images of untreated and LT-treated lung epithelial cells revealed a circular pattern of ordered actin bundles in star-like formation as well as large, distinct filamentous actin-containing protrusions (Figure 3A). As F-actin content in general was enhanced (Figure 3A, 3F), analysis of the actin depolymerization/polymerization cycle was performed. Quantification of remaining F-actin filaments after 1 hour of cytocholasin B treatment showed delayed actin depolymerization in cells exposed to LT (Figure 3B). After washout F-actin filaments repolymerized into the originally observed phenotypes in control and LT-treated cells (data not shown). Incubation of LT-treated cells with latrunculin A, which prevents actin polymerization by forming complexes with actin monomers, produced a depolymerization delay similar as illustrated for cytocholasin B (data not shown). The microtubule network and the actin cytoskeleton are interdependent, and thus profound actin reorganization is likely accompanied by changes in the microtubular system. Untreated primary lung epithelial cells displayed a radial meshwork of thin and wavy α-tubulin-containing structures. Exposure to LT produced a different pattern, where long microtubules extended straight to the cell periphery and into the large protrusions, resembling a “combed” phenotype (Figure 3C). Depolymerization of microtubules with nocodazole was delayed in toxin-treated cells as visualized by staining for stable microtubules using an antibody recognizing acetylated tubulin (Figure 3D). We also noticed increased attachment of lung epithelial cells after 24–48 hours of incubation with LT. Confocal analysis of vinculin-containing focal adhesions (FA) revealed a more elongated FA phenotype (Figure 3E), although the total number of focal contacts remained unchanged. A substantial increase of proteins connected to focal adhesions such as vinculin, paxillin and cortactin and their redistribution to the cytoskeletal fraction was observed (Figure 3F).

### MKK/MAPK inhibition mimics lethal toxin-mediated lung cell motility defects

**Figure 4:**
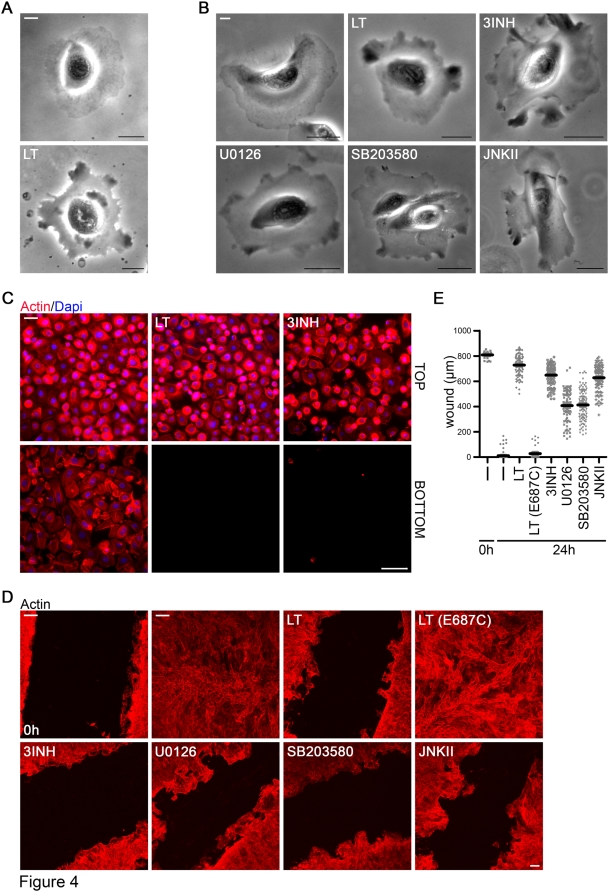
LT mediates polarization and motility defects in airway cells. (A) A representative frame of bright field live cell movies of untreated and LT-treated NHBE (48 h) after global growth factor stimulation (see Methods). Scale bar represents 20 µm. The complete movies (Movie S1 untreated and Movie S2 LT-treated) are in Supplemental data. (B) One representative frame of bright field live cell movies of untreated (-), LT-treated (48 h) or MKK/MAPK inhibitor-treated (48 h) NHBE cells after global growth factor stimulation (see Methods). Scale bar represents 20 µm. The corresponding movies (S3–8) can be found in the Supplemental part. (C) Chemotaxis of untreated (-), LT-treated (48 h) or MKK/MAPK inhibitor-treated (48 h) NHBE in Boyden chambers. 3INH is a combination of three inhibitors (U0126, SB203580, JNKII). Cells were stained with rhodamine phalloidin (red) and DAPI (blue). Upper panels depict cells on the top of the membrane after 1 h of plating before growth factors were added to the bottom chamber. The lower panels show cells on the bottom of the filter 21 h after addition of growth factors. Scale bar represents 50 µm. (D) Immunofluorescence imaging of wound healing assay using polarized NHBE (actin, red). Untreated (-) 0 h image displays the original scratch, whereas all other images were taken 24 h later. Cells were left untreated or treated with LT, catalytically inactive LT (E687C), 3INH combination, or inhibitors for MKK1/2 (U0126), p38 (SB203580) or JNK (JNKII) for 48 h before the scratch was performed. Scale bar represents 100 µm. (E) Quantification of experiment shown in (D). Data (mean+/−SEM) are represented as width of the wound of 4–8 different areas with 2 inserts/condition. The overall analysis of variance between all groups was highly significant: F_6,604_ = 675.4, p<0.0001.

The dynamic reorganization of the actin cytoskeleton regulates spatial organization of transmembrane proteins and thus plasma membrane dynamics required for polarization and directed migration. The altered physical state of LT-treated lung epithelial cells suggested distinct mechanical properties of these cells after intoxication. Time-lapse videomicroscopy of randomly migrating lung epithelial cells showed leading edge formation and persistent movement of polarized cells. After 24–48 hours of LT treatment cells remained stationary lacking microtubule organizing center (MTOC) polarization, but ruffled profoundly, exhibiting large, highly mobile protrusions, as visualized by actin and tubulin staining (Figure 4A, Movie S1, S2).

LT was efficiently taken up into lung epithelial cells grown in 2D and 3D conditions, cleaving MKK1-7 (except MKK5) in 1–2 hours as observed in other cell types. Even after 48 hours of incubation with LT, MKK cleavage was sustained and MKK proteins could not be detected (Figure S3B–C). As reported earlier in macrophages [18], this loss of MKKs is not due to reduced MKK transcription (Figure 3D). As MAPKs have been implicated in cytoskeletal dynamics and their upstream signaling partners MKKs represent the only identified LT targets, we reasoned that the striking, LT-induced cell morphology changes could be triggered by LT-mediated disabling of the Erk, JNK or p38 pathway. Inhibition of these signaling cascades can be mimicked by incubation with chemical inhibitors of the kinase activity of MKK1/2 (U0126; for downstream Erk1/2 inhibition), of JNK (JNKII) and of p38 (SB203580). All three inhibitors altered the morphological phenotype and cell motility of lung epithelial cells, although JNK inhibition changed the cell shape in a distinct manner, leading to overall cell contraction. The combined use of all three inhibitors, a treatment mimicking LT action on MAPK activity, resembled closely the LT-induced phenotype (Figure 4B, Movie S3, S4, S5, S6, S7 and S8).

Formation and orientation of a single stable protrusion together with the polarization of the MTOC is a prerequisite for directed migration. The morphology observed in lung epithelial cells after LT exposure suggested defects in cell functions, which rely on directed migration such as chemotaxis or wound healing. Using growth factors as chemotactic stimulus, complete inhibition of lung epithelial cell migration through Boyden chambers was observed when cells were treated with LT or the three inhibitor combination (Figure 4C). Both, untreated and LT-treated cells, were reseeded on fibronectin-coated filters for these experiments, since adherence on collagen or fibronectin matrix was not altered after exposure of cells to LT (Figure S3A). In all conditions attachment to the top of the filter occurred similarly well.

Polarized primary lung epithelial cell layers exhibit a rapid wound healing response where even a large wound closes in 18–22 hours. Scratch assays were performed on untreated or LT-treated epithelial layers, which were then compared to wounded 3D layers treated with MKK/MAPK inhibitors (Figure 4D, 4E). Catalytically active LT inhibited wound closure, while an inactive LT mutant (LT E687C) had no effect. Application of MKK1/2, JNK or p38 inhibitors in combination resembled the LT-induced block in wound healing, while each inhibitor by itself led only to partial inhibition.

### Role of Rho GTPases in LT-mediated motility defects

The changes in cytoskeletal and microtubule dynamics including the pronounced membrane ruffling of LT-treated cells suggested alterations in the activity of Rho GTPases. Cdc42, Rac1 and RhoA have been well established as mediators of dynamic reorganization of the actin cytoskeleton, microtubule dynamics and junction maintenance in epithelial cells [19], [20]. The distinct protrusion and stress fiber formation may indicate aberrant Rho GTPase activation by LT. Thus, cell lysates from untreated and LT-treated lung epithelial cells were probed for Rho GTPase activity using pulldown assays. While Rac1 and RhoA activity did not change, an increase in active GTP-bound Cdc42 was observed (Figure S4A). Directed migration is dependent on tight spatio-temporal control and cycling between on and off states, and could be disrupted by locking Cdc42 into the activated state. Although increased RhoA activity was not detected, we probed if inhibition of downstream effectors of Rho, ROCK-1 and MLCK, could suppress some of morphological features of LT-treated cells. The ROCK-1 inhibitor Y27632 rescued partially the LT-mediated increase of E-cadherin and the diffuse junction expansion, while the MLCK inhibitor ML-7 was ineffective (Figure S4C and data not shown). However, Y27632 did not prevent the LT-induced permeability increase in polarized 3D lung epithelium (Figure S4B). The phosphorylation status of cofilin, a ROCK-1/PAK target required for motility [21], [22], was also analyzed. Untreated and LT-treated lung epithelial cells did not differ in their phospho-cofilin content (data not shown).

### Rescue of migration defects by non-cleavable MKKs

**Figure 5:**
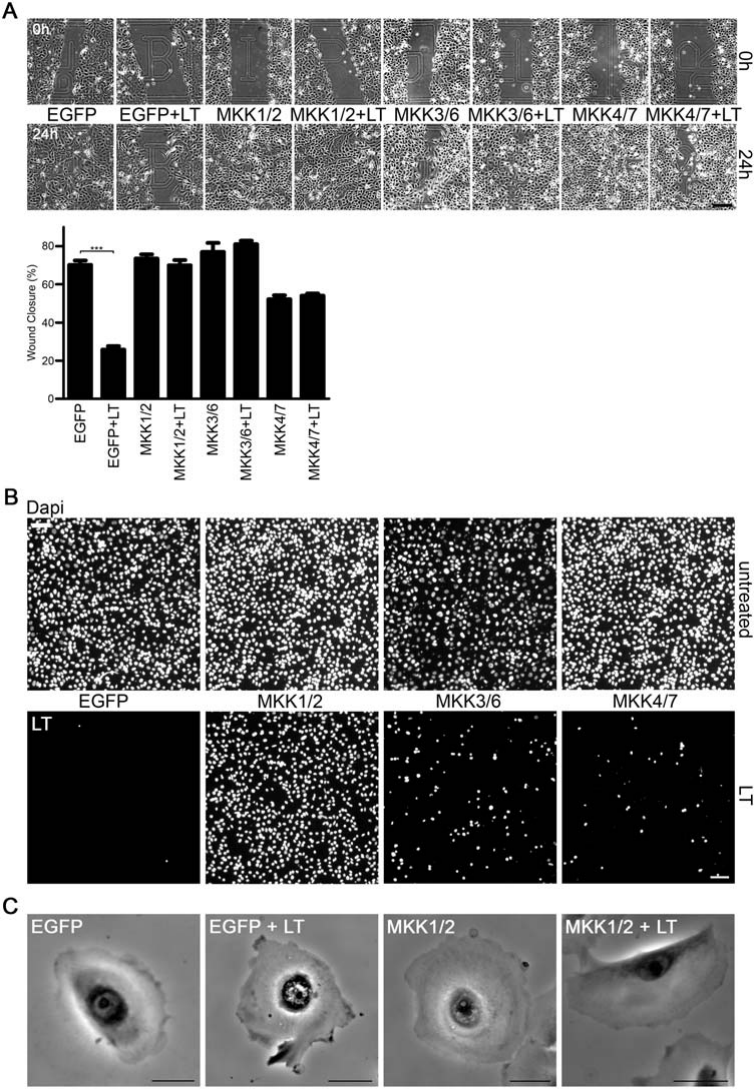
LT-mediated migration defects are MKK-dependent. (A) Live cell wound healing assay was performed in untreated or LT-treated airway cells (SALE) expressing non-cleavable MKK1/2, MKK3/6 or MKK4/7 mutants or EGFP alone as control. Cells were imaged at time zero and 24 h after the scratch. Scale bar represents 50 µm. Quantification of experiment is shown in lower panel. Data (mean+/−SEM) in each experiment were obtained from 10 different wound areas, n = 3. The overall analysis of variance between all groups was highly significant: F_7,72_ = 49.6, p<0.0001. (B) Chemotaxis of untreated and LT-treated airway cells expressing non-cleavable MKK1/2, MKK3/6 or MKK4/7 using Boyden chambers (migration for 21 h). Cells on the bottom of the filter were stained with DAPI (blue). Upper panel depicts untreated cells, lower panel LT-treated cells. Scale bar represents 50 µm. (C) A representative frame of bright field live cell movies of untreated and LT-treated airway cells expressing EGFP or non-cleavable MKK1/2-/EGFP after global growth factor stimulation. Scale bar represents 20 µm. Complete movies (S9–12) can be found in Supplemental data.

Emerging data implicate signaling by MAP kinases and MKK-containing scaffolds in cellular processes required for migration [23], [24]. As the combination of chemical inhibitors for all three MAPK pathways reproduced the LT-induced phenotype in lung epithelial cells, mutants for all six, LT-targeted MKKs were prepared. The LT cleavage sites for MKKs have been identified [25] and single or multiple mutations were introduced into wildtype MKKs to destroy these sites. Transiently transfected MKK mutants were tested for expression, resistance to LT cleavage and their ability to relay exogenous signals to specific downstream MAPKs in LT-treated cells (Figure S5A). Non-cleavable MKKs were cloned into GFP-coexpressing lentiviral vectors to virally transduce primary lung epithelial cells. Live cell imaging indicated that introduction of single MKK mutants did not rescue the LT phenotype (data not shown). In order to transduce cells stepwise with pairs of MKKs required for signaling to Erk1/2, JNK, or p38 (MKK1,2; MKK4,7; MKK3,6) selection and sorting of cells was necessary. Thus, immortalized human lung epithelial cells (SALE) were used, which have been extensively characterized in the laboratory and show many of the hallmarks of primary lung epithelial cells. SALE cells transduced with GFP in combination with non-cleavable MKKs (Figure S5B) were subjected to a wound healing assay in 2D cell culture conditions as only primary, low passage cells can differentiate to the typical multicellular, polarized epithelial layers. In these conditions, expression of all three MKK pairs permitted efficient wound closure in LT-treated cells (Figure 5A). We tested if chemotaxis in Boyden chambers would be restored by expression of non-cleavable MKKs. Only introduction of the non-cleavable MKK1,2 pair overcame the LT-induced block in directed migration (Figure 5B). Non-cleavable MKK3,6 expression slightly improved the LT-mediated inhibition of chemotaxis, but was clearly less effective. Time-lapse videomicroscopy confirmed that expression of the MKK1/MKK2 mutant pair, which cannot be altered by LT and maintains its ability to signal to MKK1,2 targets including Erk1,2, restored reorientation of the MTOC, leading edge formation and motility (Figure 5C, Movie S9, S10, S11 and S12).

### Partial rescue of barrier function by keratinocyte growth factor and dexamethasone

**Figure 6:**
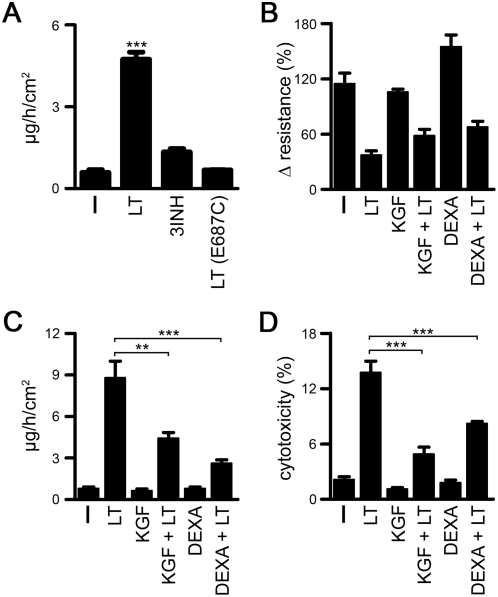
Keratinocyte growth factor and Dexamethasone partially rescue lung barrier function by reducing cell death. (A) Permeability measurements were performed in the *ex vivo* differentiated NHBE treated with or without LT (1 µg/ml LF/PA), a three inhibitor combination (3INH / U0126, SB203580, JNKII) or catalytically inactive LT (1 µg/ml LF E687C/PA) for 48 h. (B–D) Polarized NHBE layers were treated for 1 h with KGF (250 ng/ml) or dexamethasone (10 µM) before LT (1 µg/ml) was added for 48 h. Layers were analyzed for resistance (B), permeability (C) and LDH release (D). Data (A–D) are represented as mean+/−SEM of 3 inserts/condition. The overall analysis of variance in A–D between all groups was highly significant: (A) F_3,10_ = 145.6, (B) F_5,19_ = 22.6, (C) F_5,12_ = 36.4, (D) F_5,12_ = 63.3, p<0.0001.

Extensive denudation of the lung epithelium will not occur during anthrax infection. As shown, lung epithelial cells ceased to proliferate, but did not succumb to extensive cell death (Figure S2B). The LT-induced decrease in barrier function is likely caused by expulsion of single cells from the layer, which cannot be repaired due to the mechanical stiffness and motility defects caused by MKK cleavage. In accord, inhibition of MKK/MAPK signaling by chemical compounds was not pro-apoptotic, but reduced barrier integrity (Figure 6A). As LT inhibitors are not yet available and introduction of non-cleavable MKK1,2 is not a viable treatment option, a screening for barrier-protective drugs was initiated. Compounds, which have been used successfully for treatment of lung epithelial injury were tested for their ability to protect barrier integrity during LT exposure [26]–[28]. Two compounds, keratinocyte growth factor (KGF) and dexamethasone, rescued barrier function significantly (Figure 6B–C). Both compounds did not alter LT-induced changes of the cytoskeletal or microtubule network in 2D conditions (data not shown). We reasoned that if less cells would be expelled from the epithelial layer, the disruption of multicellular junctions would be reduced, even if LT-paralyzed motility had not been restored. Supporting this hypothesis, KGF and to a lesser degree dexamethasone reduced LT-mediated epithelial cytotoxicity considerably (Figure 6D), and thus seem to protect barrier integrity of the lung epithelium by extending cell survival.

## Discussion

In this study, the effect of long term exposure of lung epithelium to *B. anthracis* lethal toxin, which enters every mammalian cell type and acts by blocking intracellular MKK-MAPK signaling cascades permanently, was determined. MKK-MAPK pathways mediate crucial signals from the extracellular milieu to gene transcription, thus affecting cell proliferation, apoptosis, differentiation and inflammatory mediator release. Accumulating evidence suggests that MAPKs might also control cell migration, although the precise mechanism remains obscure [23], [24]. After 24 hours of LT exposure, complete MKK1-7 (except MKK5) deficiency was observed in lung epithelial cells, which was maintained over the observation period of up to 4 days. LT-mediated cytoskeletal alterations, present after the first 24 hours, were sustained even when a depolymerization / repolymerization cycle was induced by addition and subsequnent removal of depolymerizing agents. The presence of multiple overlapping cytoskeletal targets, which control actin and microtubule dynamics indicate that coordinated input from all three major MAPK pathways might be important for maintenance of cell shape and motility. The distinct morphological and functionally paralyzed phenotype caused by exposure of primary human lung epithelial cells to LT supports this notion. Some networks involved in motility may have redundancy, as the successful 2-dimensional wound closure by three different pairs of non-cleavable MKKs suggests. On the other hand, introduction of single non-cleavable MKKs into lung epithelial cells was not sufficient for reversal of paralyzed actin and microtubule networks. Thus, one may speculate that MKK1 and MKK2 may trigger not only Erk1 and Erk2 signaling pathways. MKKs may participate in interactions with molecules essential for connecting membrane dynamics with actin assembly such as cortactin or MP1 [24], [29]. The morphological phenotype of LT-treated lung epithelial cells showed mechanical stiffness of the cell body with increased F-actin content and redistributed focal adhesion proteins in concert with highly dynamic, randomly oriented protrusions. Upregulation of β-actin by LT was also reported by comparative proteomics studies on mouse macrophages [30]. In contrast, fMLF-stimulated F-actin filament assembly was reduced in LT-treated neutrophils [10]. The same group reported decreased actin assembly of *Listeria* rocket tails in HeLa cells exposed to LT [11], which was linked to defective actin monomer transport by Hsp27, a phosphorylation target of p38 MAPK. In our study, F-actin content was increased and introduction of non-cleavable MKKs upstream of p38 MAPK rescued 2-dimensional lung epithelial wound healing only if signaling by both, MKK3 and MKK6, was restored. This rescue was not specific for the MKK3/MKK6 pair and did not improve chemotaxis. The cytoskeletal networks of neutrophils that are required for priming responses, polarization, and chemotaxis are quite distinct, and likely controlled by different signaling pathways than those important for cytoskeletal responses and junction formation in other cell types. Endothelial cells, which also form a tight barrier, seem to undergo cell elongation after LT treatment and succumb more or less to apoptosis [14], [31]. MKK/MAPK signaling seems not to be required for all cytoskeletal remodeling processes. For example, the actin reorganization required for phagocytosis was fully maintained in bone marrow-derived murine macrophages subjected to prolonged LT treatment (strain C57Bl/6; unpublished observations).

The plasma membrane of LT-exposed lung epithelial cells showed multiple, highly active protrusions without formation of a leading edge or MTOC polarization. Development and extension of protrusions is under control of Rho GTPases. The observed enhancement of Cdc42 activity in LT-treated cells might be a redistribution effect of signaling molecules, including unidentified Cdc42 GAPs, once MKKs have been cleaved and degraded. As GTPase cycling between the on and off state is essential for protrusion formation, this increase of Cdc42 activity may cause deregulation of the Cdc42-GSK3-APC pathway [32]. Blocking GSK-3 leads to randomly oriented protrusions and inhibition of MTOC re-orientation [33]. Additionally, GSK-3-mediated phosphorylation of the focal adhesion protein paxillin is Erk-dependent [34]. Deregulation of the Cdc42-GSK-3 pathway by LT intoxication is supported by observations that GSK-3 protein levels decrease considerably in LT-treated macrophages [35]. Introduction of non-cleavable MKK1 and MKK2 into lung epithelial cells restored normal protrusion formation and migration towards a chemotactic gradient, suggesting that the MKK1/2-Erk1/2 signaling axis serves as important upstream regulator of protrusion formation.

Forming a tight barrier is one of the key functions of the lung epithelium. Assuming that our polarized lung epithelial cell model reflects the airway epithelium *in vivo*, our data suggest that this barrier will be destroyed by uptake of high LT quantities over a prolonged time during the final stage of anthrax infection. In addition, the observed cytoskeletal changes will lead to an inability to repair the epithelium. The initial damage seems not to be massive cell death and tight junction destruction, but rather expulsion of senescent single cells as observed as prerequisite for *Listeria* invasion [36]. In healthy conditions, rapid formation of new multicellular junctions will repair the damage and restore the barrier. Exposure to LT increases cytotoxicity and thus the expulsion rate, and hinders repair by MKK cleavage-induced inhibition of actin and microtubule dynamics in the neighboring lung epithelial cells. Treatment with KGF or the synthetic glucocorticoid dexamethasone alleviated LT-mediated barrier damage significantly, thus providing a potential therapeutic option for anthrax victims. Both of these agents have previously been implicated in lung epithelial repair mechanisms [37]. Although we could not confirm direct effects of these drugs on cytoskeletal dynamics, our data point to a decrease in LT-induced cytotoxicity, specifically after KGF pretreatment, and thus to enhanced survival of LT-exposed polarized lung epithelial layers. The respiratory distress of critically ill anthrax patients might be caused by LT-mediated breakdown of the respiratory barrier and influx of fluid into the lungs. Preventative therapy with KGF may strengthen the airway barrier and improve lung function.

## Materials and Methods

### Antibodies, plasmids, PCR and inhibitors

Antibodies directed against ZO-1, streptavidin-HRP, GFP (A6455), mouse/rabbit alexa fluor 488, 568, 594 were from Invitrogen (Carlsbad, CA); MKK1 (C-18), MKK2 (C-16), MKK3 (C-19), MKK4 (C-20), MKK4 (K-18), RhoGDI (A-20), RhoA, Cdc42 were from Santa Cruz (Santa Cruz, CA); gelsolin (GS-2C4), acetylated tubulin (T6793), β-actin (A2066), α-tubulin (DM1A), vinculin (hVIN-1) were from Sigma Aldrich (St. Louis, MO); p38 MAPK, phospho-p38 MAPK (Thr180/Thr182), p44/42 MAP kinase, phospho-p44/42, pSAPK/JNK (Thr183/Thr185,) SAPK/JNK (56G8), and FAK were from Cell Signaling (Danvers, MA); paxillin, E-cadherin, MKK2, and Rac1 from BD Biosciences (Franklin Lakes, NJ); MKK6 from Biolegend (San Diego, CA); MKK7 (ab18565) was from Abcam (Cambridge, MA); cortactin (p80/85 clone 4F11) from Millipore; MKK1 (N-term) from Chemicon (Temecula, CA). Mammalian expression vectors containing MKK3 (I27E) and MKK6 (I15A) were from Michael David (UCSD), MKK1wt and MKK2wt from Kun-Liang Guan (U. of Mich.), and pcDNA3 MKK7wt was provided by Jiahuai Han (TSRI). pBluescriptR MKK4wt was from Open Biosystems (Huntsville, AL) (clone ID 5272439). Point mutations for MKK1 (P8A), MKK2 (P10A), MKK4 (K45Q, K46E, R58Q, F59D) and MKK7 (Q44K, G76A) were introduced by QuickChange® Site-Directed Mutagenesis Kit (Stratagene, La Jolla, CA) and verified by sequencing. Mutant MKKs were cloned into the lentiviral vector CGW-IRES-EGFP. The CGW vector and the lentiviral plasmids for GAG/pol, VSV-G and pRSV-rev were provided by Bruce Torbett (TSRI). Conditions and primers for RT-PCR are described in Methods S1. Inhibitors for p38 (SB203580, 10 µM; SB202190, 10 µM), MKK1/2 (U0126, 5 µM), JNK (JNKII, 5 µM), ROCK-1 (Y27632, 10 µM) and dexamethasone (10 µM) were purchased from EMD Biosciences (San Diego, CA). Cytocholasin B (1 µg/ml), nocodazole (10 nM), MTT (0.5 mg/ml), DAPI (1 µg/ml), PMA (100 ng/ml), fibronectin (10 µg/ml), human placenta collagen type VI (10 µg/ml) and rhodamine phalloidin were from Sigma Aldrich (St. Louis, MO). EF was from List Biological Laboratories, Inc. (Campbell, CA). LF (E687C) was a gift from Nick Duesbery (Van Andel Research Institute, MI) and keratinocyte growth factor (250 ng/ml) was purchased from Cell Sciences (Canton, MA). TNFα was from Thermo Fisher Scientific Inc. (25 µg/ml, Rockford, IL). Expression and purification of PA and LF is described in Methods S2.

### Cell culture

Normal human bronchial epithelial cells (NHBE) were purchased from Lonza (Walkersville, MD) and used at passage 3–5. SALE cell were a gift of W. C. Hahn [38]. Cells were grown in serum-free medium (SABM, Lonza) supplemented with SAGM SingleQuots (Lonza). A polarized lung epithelial model (3D) was established by seeding NHBE cells onto inserts with semi-permeable support membranes (Transwell culture insert, 0.4 µm pore, Corning, Lowell, MA) coated with human placenta collagen type VI (15 µg/cm^2^, Sigma Aldrich, St. Louis, MO). For differentiation, airway epithelial cells were grown in 50% SABM, 50% DMEM high glucose supplemented with SingleQuots and 50 nM retinoic acid. Cells were grown in air-liquid interface for 3–4 weeks. HeLa and HEK293T cells were cultured in DMEM high glucose plus 10% FBS. If not otherwise indicated, cells were starved for 3 h and treated with 1 µg/ml of PA, LF and/or EF for 24–48 h in media without supplements.

### Lentivirus production and transduction

Non-cleavable MKK mutants were cloned into a bicistronic CGW-IRES-EGFP vector [39]. Transfection of HEK293T cells was with calcium chloride (10 µg transfer vector plasmid, GAG/pol plasmid, VSV-G plasmid, pRSV-rev plasmid) in DMEM high glucose. Virus suspension was filtered and concentrated by ultracentrifugation at 19400 rpm for 2.5 hours. SALE cells were transduced with non-cleavable MKK mutants, sorted for medium EGFP expressing cells by flow cytometry and consecutively transduced with a second non-cleavable MKK mutant (FACS Vantage DiVa, BD Biosciences, San Jose, CA). MKK and GFP expression levels of stable SALE cell lines were verified by immunoblotting.

### Immunofluorescence microscopy

NHBE cells were seeded on a glass coverslips coated with human placenta collagen type VI (10 µg/ml). At 50–80% confluency cells were fixed with 4% paraformaldehyde (actin, vinculin), ice cold methanol (acetylated tubulin) or methanol/acetone (ZO1, E-cadherin). After rehydration in PBS, permeabilization with 0.5% Triton X-100 and blocking with 5% BSA in PBS, coverslips were incubated for 1 h with primary antibody in 2% BSA/PBS. The secondary antibodies were anti-rabbit or anti-mouse IgG conjugated with alexa-fluor-488 or -568 (Invitrogen, Carlsbad, CA). All images were taking by confocal microscopy (20× or 63×, MRC 1024, BioRad, Hercules, CA) with the same laser power, gain and offset per antibody condition.

### Migration assay in Boyden chambers

After LT treatment (1 µg/ml PA and LF) cells were reseeded in SABM media without supplements on polycarbonate membrane filters coated with fibronectin (pore size 8 µm). Migration occurred towards SABM with SingleQuot supplements in the bottom compartment for 21 h. Cells on top of the filter were removed, while cells on the bottom of the filter were fixed with PFA and stained with rhodamine phalloidin and DAPI. Images were taken with an Axioskop microscope (Zeiss, Goettingen, Germany) by using the AxioVision AC 4.2. software. Pictures were analyzed with ImageJ (NIH, Bethesda, MD).

### Wound healing assay

NHBE differentiated for 3 weeks and treated for 48 h with or without LT and/or inhibitors were wounded using a pipette tip. The cells were either fixed immediately as control or incubated for 24 h and then fixed. After staining with rhodamine phalloidin confocal images with a 10× objective lens were taken (MRC 1024, BioRad, Hercules, CA). ImageJ (NIH, Bethesda, MD) was used to analyze the width of the scratch. Two different inserts per condition and 6–8 images with 10 measurements per image were used. For wounding assays in normal cell culture conditions SALE cells expressing non-cleavable MKK mutants were plated on human collagen-coated, etched grid coverslips (Bellco, Vineland, NJ) and grown to 100% confluency. Cells were left untreated or treated for 24 h with LT before wounding. Images of wounded cells were taken immediately and 24 h later after incubation in SABM media with supplements (Olympus IX70 microscope; Olympus, Center Valley, PA).

### Live cell imaging

A Nikon Eclipse TE2000-U (Melville, NY) was used to generate movies of untreated or treated NHBE or SALE cells. Cells were plated on collagen-covered cover slips, grown for two days, starved in SABM media for 3 hours and treated with 1 µg/ml PA and 1 µg/ml LF for 48 h (NHBE) or 24 h (SALE) prior to imaging. The cells were washed with HBSS before SABM media containing SingleQuot supplements (growth factors) was added just before live cell imaging started. Bright field images were taken every 30–60 sec for 40–120 min with a 20× objective lens. Metamorph (Molecular Devices Corp., Downingtown, PA) or ImageJ (NIH, Bethesda, MD) were used to analyze the pictures and generate movies.

### Transmission electron microscopy (TEM)

TEM of untreated and LT-treated differentiated NHBE was performed as described [40].

### Western blot analysis

Proteins were extracted in RIPA buffer (150 mM NaCl, 50 mM Tris-HCl, pH 7.4, 0.5% sodium cholate, 1% NP-40, 0.1% SDS, protease and phosphatase inhibitor cocktail (Roche, Indianapolis, IN). SDS-PAGE was performed and the proteins were transferred onto nitrocellulose membranes. Membranes were blocked with 5% BSA, incubated with specific and HRP-coupled secondary antibodies. Bands were visualized using ECL (Thermo Scientific, Rochford, IL).

### Cell fractionation

NHBE cells were lyzed in fractionation buffer (25 mM Tris, pH 7.5, containing 2 mM EDTA, pH 8, 10 mM β-mercaptoethanol, 10% glycerol, 10 µg/ml leupeptin, 10 µg/ml aprotinin, 1 mM phenylmethylsulfonyl fluoride). The post-nuclear supernatant (1000×g for 10 minutes) of sonicated cells was spun at 100,000×g for 30 minutes in an Optima TLX ultracentrifuge (Beckman Instruments Inc., Palo Alto, CA). The supernatant (cytosol) was frozen und the pellet was washed with fractionation buffer. After incubation of the pellet on ice for 45 minutes in fractionation buffer containing 1% (v/v) Triton X-100, triton-soluble membranes were separated from the triton-insoluble cytoskeletal fraction by centrifugation at 100,000×g for 30 minutes. The cytoskeletal pellet was washed and sonicated in an equal amount of fractionation buffer.

### GTPase activity assay

PBD pulldown assay for detection of Rac and/or Cdc42 activity, and the rhotekin Rho binding domain-based assay for RhoA activity were performed like previously described [41], [42].

### Cell viability assays

MTT assay for cells grown in 2D: MTT (500 µg/ml) was added to cells for 30 min or 1 h. After PBS wash the water-insoluble purple formazan was dissolved in DMSO (Sigma Aldrich, St. Louis, MO). Absorbance at 560 nm was determined for each well using a microplate reader (Bio-Rad, Hercules, CA). LDH release for cells grown in 3D: LDH was determined using the CytoTox-ONE™ Kit from Promega (Madison, WI). The fluorescence was measured with an excitation wavelength of 560 nm and an emission wavelength of 590 nm in a Synergy HT fluorimeter (Bio-Tek Instrument Inc., Winooski, VT). Cytotoxicity (%) was calculated as follows: Cytotoxicity (%) = 100×(amount of LDH in basal medium minus background) / (amount of LDH of lysed apical cells minus background).

### Adhesion assay

NHBE were grown for 2 days, starved for 3 h in SABM media, and treated with 1 µg/ml PA and LF for 48 h. 24well plates were coated with fibronectin (10 µg/ml) or collagen (5 µg/ml) and cells were seeded in triplicates. After 24 h cells were washed carefully and adherent cells were stained with 500 µg/ml MTT.

### Resistance measurements

Transepithelial electric resistance (TER) was measured by using a voltohmmeter (EVOM) with STX2 electrode (World Precision Instruments, Sarasota, FL).

### Permeability measurements

FITC-coupled albumin (0.5 mg/ml in HBSS, A7016, Sigma Aldrich, St. Louis, MO) was applied to the apical side of differentiated NHBE. Leakage of the dye through the epithelial layer was measured in samples removed from the bottom chamber using an excitation wavelength of 485±20 nm and an emission wavelength of 530±25 nm. The standard curve equation of x = y/15.195 calculates the permeability of the cell layer in µg. The time period of incubation and the insert surface was taken into account for final calculation (µg/h/cm^2^).

### Statistical analysis

Data are represented as mean±SEM. Figure 1 was performed with unpaired two-tailed t-tests. Significance levels were *, *P*<0.05; **, *P*<0.01; ***, *P*<0.001. Overall comparison among groups within Figure 4, 5, 6, S2, S4 was analyzed with one way analysis of variance. If the overall F statistics from the one-way ANOVAs were significant at the alpha = 0.05 level, paired group comparisons were performed with a Bonferroni correction for multiplicity. Significance levels were *, *P*<0.05; **, *P*<0.01; ***, *P*<0.001.

## Supporting Information

Methods S1 (0.03 MB DOC)

Methods S2 (0.04 MB DOC)

Figure S1 Characterization of the polarized airway system and expression of anthrax receptors in NHBE cells. (A) TEM image of cross section obtained from untreated lung epithelial layers ALI-cultured for 5 weeks. A crosscut with apical cilia, different cell types including goblet cells, tight junction formation and cell interdigitations is depicted. Scale bar represents 5 µm. (B) Expression of differentiation markers caveolin 1, human defensins 1 (hBD-1) and 2 (hBD-2) in cell culture-plated cells (2D) versus polarized airway system (3D) (RT-PCR). Actin served as loading control. (C) Expression of surfactant A and B (SP-A, SP-B) in differentiated lung epithelial layers (RT-PCR of 3D, see B). PCR bands were not detected in 2D conditions (not shown). (D) Increased MUC5AC expression in polarized lung epithelium (RT-PCR). Actin was used as loading control. (E) Expression of anthrax receptors ATR/TEM8 and CMG2 in NHBE 2D (RT-PCR). Actin served as loading control.
(1.34 MB TIF)

Figure S2 LT exposure leads to permeability increase and proliferation inhibition in NHBE. (A) The epithelial layer was incubated with FITC-albumin on the apical side for permeability measurements after no, LT, ET and LT+ET treatment (48 h). One representative experiment in triplicate is shown (data are represented as mean+/−SEM). The overall analysis of variance between all groups was highly significant: F_3,8_ = 397.4, p<0.0001. (B) Untreated or LT-treated NHBE (48 h) were washed and incubated with LT-free media containing growth factors for additional 48 h (48+48) or 96 h (48+96). Metabolically active cells were measured using the MTT assay. Data are represented as mean+/−SEM of 3 independent experiments.
(0.10 MB TIF)

Figure S3 Adhesion parameters and MKK expression profiles after LT-induced MKK cleavage in NHBE cells. (A) LT-treated (48 h) and untreated NHBE cells were reseeded on fibronectin- or collagen-coated dishes. Number of cells attached after 21 h was quantified using the MTT assay. Data are represented as mean+/−SEM of 3 different experiments. (B) Immunoblot depicting MKK cleavage after LT treatment (48 h) of NHBE cells grown in cell culture conditions (2D). Actin served as loading control. (C) Immunoblot depicting MKK cleavage in polarized NHBE cultures (3D). Actin served as loading control. (D) Expression of MKK2 and MKK3 in NHBE treated with PA or LT (RT-PCR). Actin served as loading control. First lane represents no template control.
(0.37 MB TIF)

Figure S4 Rho GTPase activity and effect of ROCK-1 inhibition on junctions. (A) Pulldown assays for Cdc42-GTP, Rac1-GTP and RhoA-GTP with or without LT treatment for 48 h. Active, bound Rho GTPases were detected by immunoblot. Total cell lysate (TCL) was used as loading control (5% of total). (B) Permeability of polarized NHBE layers after no treatment, LT, Y27632 and Y27632+LT treatment for 48 h. One representative experiment in triplicate is shown (data are represented as mean+/−SEM). The overall analysis of variance between all groups was highly significant: F_3,8_ = 416.6, p<0.0001. (C) NHBE cells were treated with or without LT, Y27632 and Y27632+LT and stained for E-cadherin (green). Confocal images were taken consecutively. Scale bar represents 50 µm.
(3.00 MB TIF)

Figure S5 Validation of non-cleavable MKKs. (A) Non-cleavable MKK mutants or empty vector plasmid were transiently transfected into HeLa cells and expressed for 24 h followed by 24 h of LT treatment. Cells were then stimulated for 15 min with PMA (100 ng/ml) or TNFα (25 µg/ml) to activate MKK/MAPK pathways. Endogenous MKK expression; exogenous, non-cleavable MKK expression; LT-mediated MKK cleavage; phosphorylation of MAP kinases Erk, p38 or JNK; and total Erk, p38 and JNK expression are visualized by immunoblot as indicated. (B) Lentiviral expression of non-cleavable MKK pairs (MKK1/2, MKK3/6, MKK4/7) together with EGFP and of EGFP alone in SALE cells (see Methods). Lysates of the four SALE cell lines were probed by immunoblot with MKK specific and GFP antibodies.
(1.45 MB TIF)

Movie S1 Random migration of untreated NHBE cells. Time-lapse microscopy of untreated NHBE cells after global growth factor stimulation. The movie shows bright field images taken every minute over a time period of 2 h. The scale bar represents 20 µm.
(2.49 MB AVI)

Movie S2 Inhibition of polarization and exaggerated protrusions in LT-treated NHBE cells. Time-lapse microscopy of LT-treated NHBE cells (48 h) after global growth factor stimulation. The movie shows bright field images taken every minute over a time period of 2 h. The scale bar represents 20 µm.
(2.64 MB AVI)

Movie S3 Motility of untreated NHBE cells. Time-lapse microscopy of untreated NHBE cells after global growth factor stimulation. The movie shows bright field images taken every 30 seconds over a time period of 40 minutes. The scale bar represents 20 µm.
(9.58 MB AVI)

Movie S4 Inhibition of motility in NHBE cells treated with LT. Time-lapse microscopy of LT-treated (48 h) NHBE cells after global growth factor stimulation. The movie shows bright field images taken every 30 seconds over a time period of 40 minutes. The scale bar represents 20 µm.
(7.10 MB AVI)

Movie S5 Inhibition of motility in NHBE cells treated with a triple MKK/MAPK inhibitor combination. Time-lapse microscopy of NHBE cells incubated with inhibitors U0126, SB203580 and JNKII (48 h) after global growth factor stimulation. The movie shows bright field images taken every 30 seconds over a time period of 40 minutes. The scale bar represents 20 µm.
(10.12 MB AVI)

Movie S6 Inhibition of motility in NHBE cells treated with MKK1/2 inhibitor. Time-lapse microscopy of NHBE cells incubated with inhibitor U0126 (48 h) after global growth factor stimulation. The movie shows bright field images taken every 30 seconds over a time period of 40 minutes. The scale bar represents 20 µm.
(7.09 MB AVI)

Movie S7 Inhibition of motility in NHBE cells treated with p38 inhibitor. Time-lapse microscopy of NHBE cells incubated with inhibitor SB203580 (48 h) after global growth factor stimulation. The movie shows bright field images taken every 30 seconds over a time period of 40 minutes. The scale bar represents 20 µm.
(4.69 MB AVI)

Movie S8 Inhibition of motility in NHBE cells treated with JNK inhibitor. Time-lapse microscopy of NHBE cells incubated with inhibitor JNKII (48 h) after global growth factor stimulation. The movie shows bright field images taken every 30 seconds over a time period of 40 minutes. The scale bar represents 20 µm.
(3.56 MB AVI)

Movie S9 Motility of SALE cells expressing EGFP. Time-lapse microscopy of untreated SALE cells expressing EGFP after global growth factor stimulation. The movie shows bright field images taken every 30 seconds over a time period of 40 minutes. The scale bar represents 20 µm.
(6.82 MB AVI)

Movie S10 Time-lapse microscopy of LT-treated (24 h) SALE cells expressing EGFP after global growth factor stimulation. The movie shows bright field images taken every 30 seconds over a time period of 40 minutes. The scale bar represents 20 µm.
(5.57 MB AVI)

Movie S11 Motility of SALE cells expressing non-cleavable MKK1/2 mutants. Time-lapse microscopy of untreated SALE cells expressing non-cleavable MKK1/2 mutants after global growth factor stimulation. The movie shows bright field images taken every 30 seconds over a time period of 40 minutes. The scale bar represents 20 µm.
(4.37 MB AVI)

Movie S12 Rescue of motility by non-cleavable MKK1/2 mutants in SALE cells treated with LT. Time-lapse microscopy of LT-treated (24 h) SALE cells expressing non-cleavable MKK1/2 mutants after global growth factor stimulation. The movie shows bright field images taken every 30 seconds over a time period of 40 minutes. The scale bar represents 20 µm.
(9.71 MB AVI)
