# Supplementary material for: Lung Epithelial Injury by B. Anthracis Lethal Toxin Is Caused by MKK-Dependent Loss of Cytoskeletal Integrity
Source: PLoS One. 2009 Mar 9;4(3):e4755. doi: 10.1371/journal.pone.0004755 (PMC2649448; doi:10.1371/journal.pone.0004755)
Supplement: Methods S1 — (0.03 MB DOC) [file pone.0004755.s001.doc]

**Methods S1**

**RT-PCR**

RNA was extracted from NHBE cells using the RNeasy kit (QIAGEN, Valencia, CA). 2μg of RNA were synthesized to complementary DNA with SuperScript^TM^ II Reverse Transcriptase (Invitrogen, Carlsbad, CA) according to protocol. The following primers were used: β-actin forward 5’-TGACGGGGTCACCCACACTGTGCCCA-3’ reverse 5’-CTAGAAGCATTTGCG GTGGACGATGG-3’ (661bp, 60°C, 18 cycles); ATR forward 5’-CCAGGAGGAGACACTTAC ATG-3’, reverse 5’-TGAGTGGATGATGCCTTGC-3’ (302bp, 50°C, 35 cycles); caveolin 1 forward 5’-TCAACCGCGACCCTAAACACC-3’, reverse 5’- TGAAATAGCTCAGAAGAGA CAT-3’ (562bp, 60°C); CMG2 forward 5’-CCTTTGATCTCTACTTCGTCC-3’, reverse 5’-GCTGTGACAATTAATGATCCT-3’ (810bp, 50°C); human defensins-1 (hBD-1) forward 5’-CTCTGTCAGCTCAGCCTC-3’, reverse 5’-CTTGCAGCACTTGGCCTTCCC-3’ (272bp, 66°C); human defensins-2 (hBD-2) forward 5’-CCAGCCATCAGCCATGAGGGT-3’, reverse 5’- GGAGCCCTTTCTGAAT CCGCA-3’ (254bp, 66°C); MKK2 forward 5’-CGTACCTC CGAGAGAAGCAC-3’, reverse 5’-GAAGGTGTGGTTTGTGAGCA-3’ (560bp, 60°C); MKK3 forward 5’-TTGGACAG GTCAGCAGACAG-3’, reverse 5’-TCCAGA GCACTCACCTCCTT-3’ (539bp, 53°C); MUC5AC forward 5’-TCCGGCCTCATCTTCTCC-3’, reverse 5’-ACTTGGGCACTGGT GCTG-3’ (662bp, 55°C); Surfactant A (SP-A) forward 5’-GAGAGATGGTATCAAA GGAG-3’, reverse 5’-GGTACCAGTTGGTGTAGTT-3’ (499bp, 58°C); Surfactant B (SP-B) forward 5’-TTCTGGTGCCAAAGCCTGGA-3’, reverse 5’-CAGGCCTGGTGCATTG CC-3’ (836bp, 58°C).
