# Supplementary material for: Lung Epithelial Injury by B. Anthracis Lethal Toxin Is Caused by MKK-Dependent Loss of Cytoskeletal Integrity
Source: PLoS One. 2009 Mar 9;4(3):e4755. doi: 10.1371/journal.pone.0004755 (PMC2649448; doi:10.1371/journal.pone.0004755)
Supplement: Methods S2 — (0.04 MB DOC) [file pone.0004755.s002.doc]

**Methods S2**

**Expression and purification of protective antigen (PA)**

PA was expressed and purified from a *Bacillus megaterium* protein expression system that also expressed the *pagA*-transcriptional activator AtxA. First, the *atxA* coding region and its promoter were PCR amplified from genomic DNA of the *B. anthracis* Sterne strain 34F2 (pXO1^+^, pXO2^-^) using oligonucleotide primers AtxA5’promEco (5’-TATAAGAATTCTATGTTAATATGCTA-3’) and AtxA3’Bam (5’-CAAATGGATCCAGGG

CATTTATATTATC-3’). The 1.6kb fragment was digested with EcoRI and BamHI and cloned in similarly digested pHT315, a replicative shuttle vector [1] generating plasmid pAtx1. The *pagA* coding sequence and promoter region were then amplified by PCR from genomic DNA of the strain 34F2 using the oligonucleotide primers BaPA5’Sal (5’-AATTAGTCGACTTTAGCTTTCTGTA-3’) and Bapagfull3’Bam (5’-TCAAAGGATCCTGA

TGATTTAGATTAGTC-3’); the fragment was cloned in the SalI-BamHI sites of plasmid pAtx1. The resulting plasmid, pHT-ATXPA, was introduced in *B. megaterium* MS941 (△nprM) [2] by the protoplast transformation method [3] selecting for erythromycin (5μg/ml) and lincomycin (25μg/ml) resistance. One colony of *B. megaterium* carrying pHT-ATXPA was grown overnight in LB medium modified to contain 35g/l of tryptone and erythromycin/lincomycin as above. Six 1 L Fernbach flasks of LB-modified medium were inoculated each with 10ml of overnight culture. Cells were grown for 14 hours at 37°C and the cells were pelleted by centrifugation at 13,000g for 30 minutes at 4°C. Cell pellet was discarded and the supernatant was kept in ice in 3 Fernbach flasks. Ammonium sulfate was added slowly to 70% saturation with gentle stirring. After at least 2 hours at 4°C without stirring, the supernatant was centrifuged at 13,000g for 1 hour at 4°C. The supernatant was removed and the protein pellet was resuspended in a total of 160ml of Buffer A for Q-Sepharose chromatography (50mM Tris-HCl pH 8.0, 50mM NaCl, 5mM EDTA). The suspension was dialyzed against 4 L of Buffer A for Q-Sepharose. The dialyzed protein was then loaded onto the Q-Sepharose column (10ml). PA did not bind to this column and was collected in the flow through but this step increased the stability of the protein. AmSO_4_ was added with gentle swirling to the flow through to 1.5M final concentration. The solution was then loaded on a Phenyl-Sepharose column pre-equilibrated with Buffer A for HI (Hydrophobic interaction) chromatography (50mM Tris-HCl pH 8.0, 1.5M AmSO_4_, 5mM EDTA). The column was washed extensively with Buffer A for HI and the protein was eluted with a linear gradient from 1.5M to 0.0M AmSO_4_. Fractions containing PA were pooled and dialyzed against Buffer A for GF (Gel Filtration) (50mM Tris-HCl pH 8.0, 150mM NaCl, 5mM EDTA). The sample was loaded onto a 60ml Sepharyl-S200HR column equilibrated with Buffer A-GF. The column was run at 0.1ml.min and 5ml fractions were collected. Fractions were analyzed by SDS-PAGE and the ones containing PA were pooled, concentrated and dialyzed against Buffer B (50mM Tris-HCl pH 8.0, 150mM NaCl, 50μM CaCl_2_). The protein was stored at -80°C in quick-frozen aliquots. The final yield was approximately 0.2mg/l of culture.

**Expression and purification of lethal factor (LF)**

LF was purified from culture supernatant of *B. anthracis* strain BH441 carrying plasmid pSJ 115 (kindly provided by S. Leppla, NIAID). Cells were inoculated in 2 Fernbach flasks containing 500ml of modified FA medium [4] supplemented with 10μg/ml kanamycin and grown for 14-16 hours at 37°C. Cultures were centrifuged, the cell pellet discarded and AmSO4 was slowly added to the supernatant with gentle stirring to reach 70% saturation. Proteins were allowed to precipitate overnight at 4°C without stirring. Proteins were collected by centrifugation at 13,000g for 1 hour. Pellet was resuspended in Buffer B-HIC for Phenyl-Sepharose Hydrophobic column (50mM Tris-HCl pH 8.0, 5mM EDTA). Solid AmSO_4_ was added to reach 1.5M final concentration and the solution was loaded onto an 8ml Phenyl-Sepharose column. After extensive washing with Buffer A-HIC (50mM Tris-HCl pH 8.0, 1.5M AmSO_4_, 5mM EDTA) the proteins were eluted with a 1.5M – 0.0M AmSO_4_ linear gradient in Buffer B-HIC. Fractions containing LF were collected and dialyzed in Buffer A for QAE (50mM Tris-HCl pH 8.0, 50mM NaCl, 5mM EDTA) and loaded onto a QAE-Sepharose column. Elution was carried out with a 50-300mM NaCl gradient. Fractions containing LF were pooled, concentrated and the buffer exchanged with storage buffer (50mM Tris-HCl pH 8.0, 50mM NaCl, 100μM ZnSO_4_, 20% glycerol). The protein was stored at -80°C. The final yield was approximately 60mg/l of culture.

References

1. Arantes O, Lereclus D (1991) Construction of cloning vectors for Bacillus thuringiensis. Gene 108: 115-119.

2. Wittchen KD MF (1995) Inactivation of the major extracellular protease from Bacillus megaterium DSM319 by gene replacement. Applied microbiology and biotechnology 42: 871-877.

3. Puyet A, H. Sandoval, P. Lopez, A. Aguilar, J. F. Martin, and M. Espinosa (1987) A simple medium for rapid regeneration of Bacillus subtilis protoplasts transformed with plasmid DNA. FEMS Microbiol Lett 40: 1-5.

4. Park S, Leppla SH (2000) Optimized production and purification of Bacillus anthracis lethal factor. Protein Expression & Purification 18: 293-302.
