# Supplementary figures and images for: Lung Epithelial Injury by B. Anthracis Lethal Toxin Is Caused by MKK-Dependent Loss of Cytoskeletal Integrity
Source: PLoS One. 2009 Mar 9;4(3):e4755. doi: 10.1371/journal.pone.0004755 (PMC2649448; doi:10.1371/journal.pone.0004755)

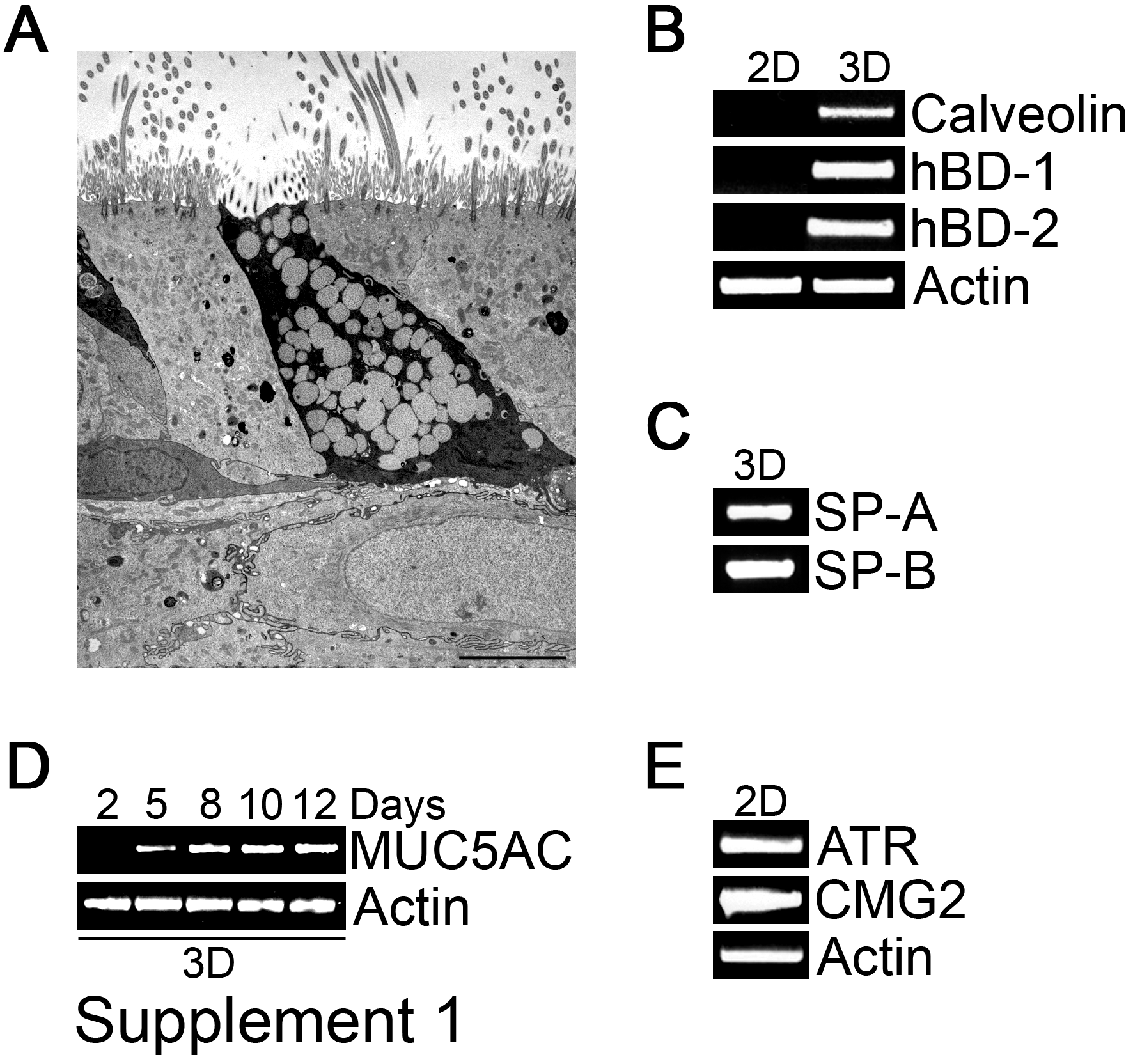

Supplement: Figure S1 — Characterization of the polarized airway system and expression of anthrax receptors in NHBE cells. (A) TEM image of cross section obtained from untreated lung epithelial layers ALI-cultured for 5 weeks. A crosscut with apical cilia, different cell types including goblet cells, tight junction formation and cell interdigitations is depicted. Scale bar represents 5 µm. (B) Expression of differentiation markers caveolin 1, human defensins 1 (hBD-1) and 2 (hBD-2) in cell culture-plated cells (2D) versus polarized airway system (3D) (RT-PCR). Actin served as loading control. (C) Expression of surfactant A and B (SP-A, SP-B) in differentiated lung epithelial layers (RT-PCR of 3D, see B). PCR bands were not detected in 2D conditions (not shown). (D) Increased MUC5AC expression in polarized lung epithelium (RT-PCR). Actin was used as loading control. (E) Expression of anthrax receptors ATR/TEM8 and CMG2 in NHBE 2D (RT-PCR). Actin served as loading control. (1.34 MB TIF) [file pone.0004755.s003.tif]

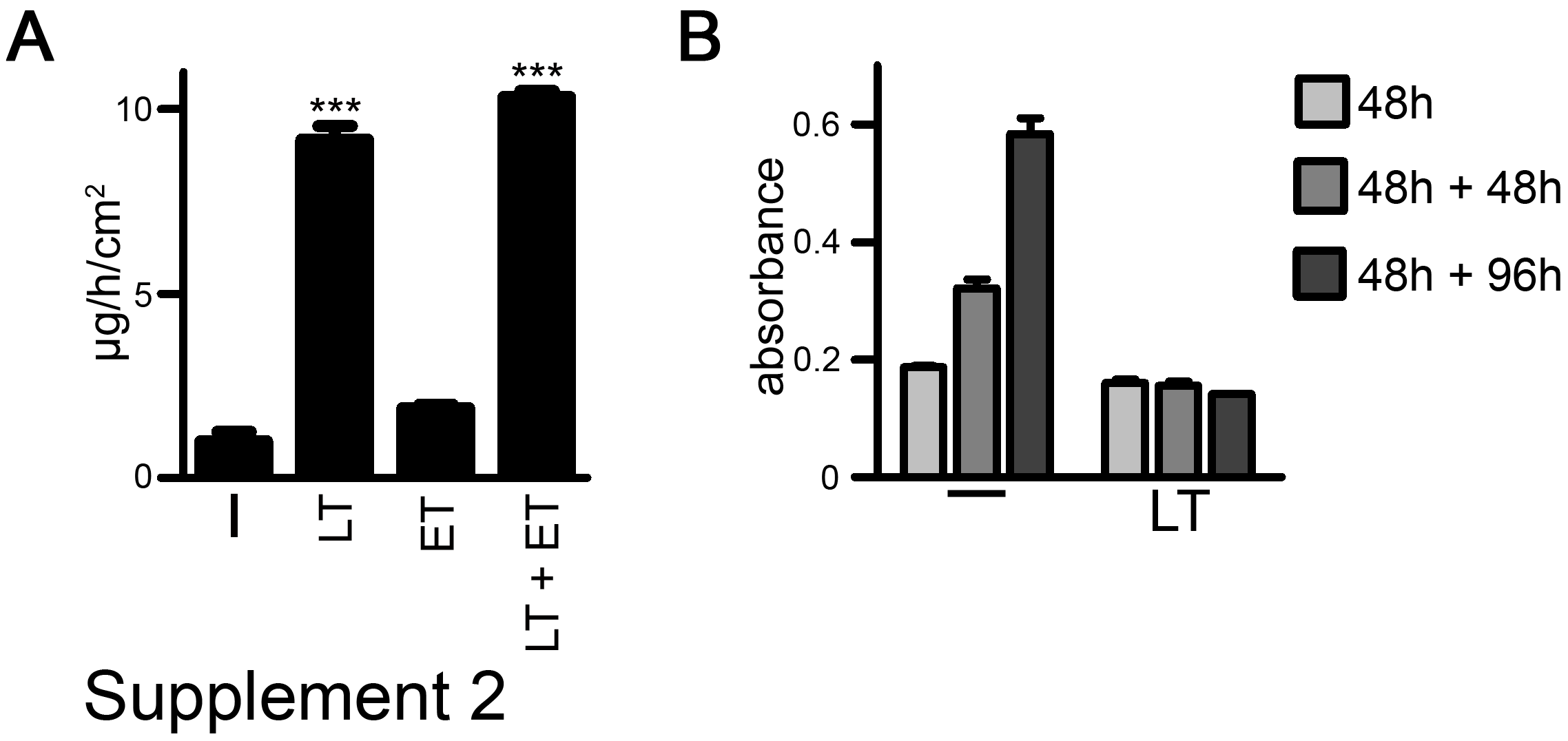

Supplement: Figure S2 — LT exposure leads to permeability increase and proliferation inhibition in NHBE. (A) The epithelial layer was incubated with FITC-albumin on the apical side for permeability measurements after no, LT, ET and LT+ET treatment (48 h). One representative experiment in triplicate is shown (data are represented as mean+/−SEM). The overall analysis of variance between all groups was highly significant: F3,8 = 397.4, p<0.0001. (B) Untreated or LT-treated NHBE (48 h) were washed and incubated with LT-free media containing growth factors for additional 48 h (48+48) or 96 h (48+96). Metabolically active cells were measured using the MTT assay. Data are represented as mean+/−SEM of 3 independent experiments. (0.10 MB TIF) [file pone.0004755.s004.tif]

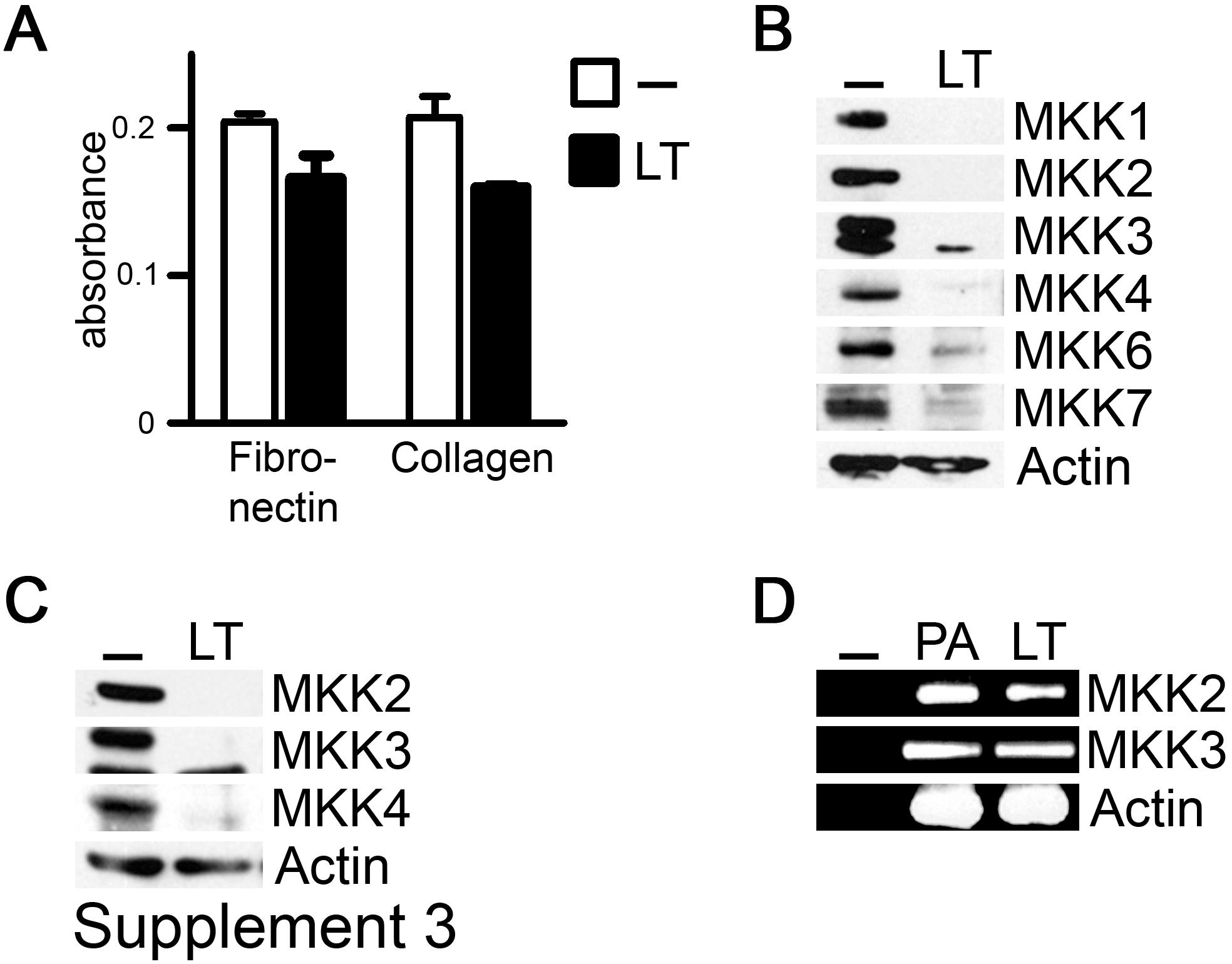

Supplement: Figure S3 — Adhesion parameters and MKK expression profiles after LT-induced MKK cleavage in NHBE cells. (A) LT-treated (48 h) and untreated NHBE cells were reseeded on fibronectin- or collagen-coated dishes. Number of cells attached after 21 h was quantified using the MTT assay. Data are represented as mean+/−SEM of 3 different experiments. (B) Immunoblot depicting MKK cleavage after LT treatment (48 h) of NHBE cells grown in cell culture conditions (2D). Actin served as loading control. (C) Immunoblot depicting MKK cleavage in polarized NHBE cultures (3D). Actin served as loading control. (D) Expression of MKK2 and MKK3 in NHBE treated with PA or LT (RT-PCR). Actin served as loading control. First lane represents no template control. (0.37 MB TIF) [file pone.0004755.s005.tif]

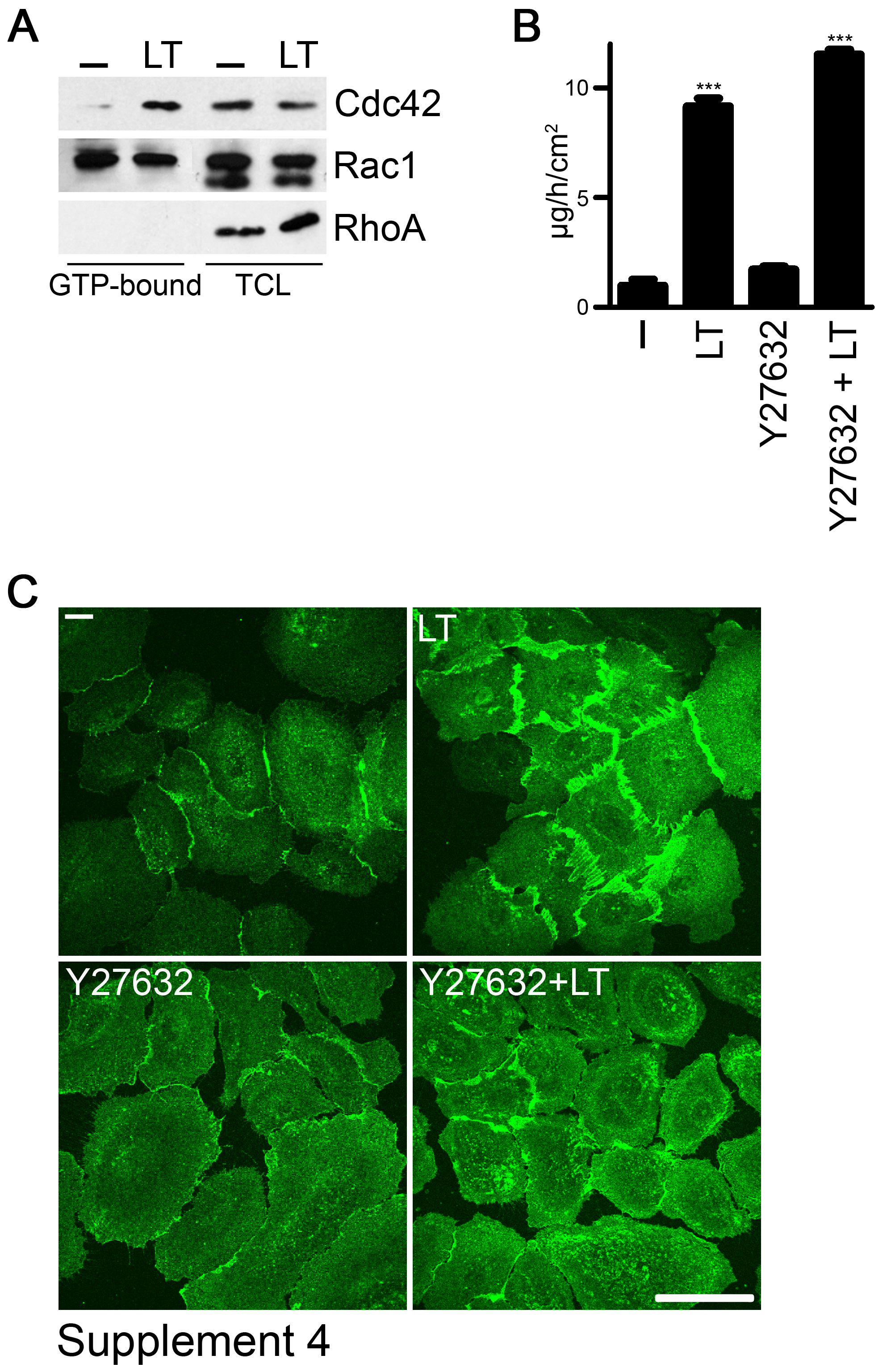

Supplement: Figure S4 — Rho GTPase activity and effect of ROCK-1 inhibition on junctions. (A) Pulldown assays for Cdc42-GTP, Rac1-GTP and RhoA-GTP with or without LT treatment for 48 h. Active, bound Rho GTPases were detected by immunoblot. Total cell lysate (TCL) was used as loading control (5% of total). (B) Permeability of polarized NHBE layers after no treatment, LT, Y27632 and Y27632+LT treatment for 48 h. One representative experiment in triplicate is shown (data are represented as mean+/−SEM). The overall analysis of variance between all groups was highly significant: F3,8 = 416.6, p<0.0001. (C) NHBE cells were treated with or without LT, Y27632 and Y27632+LT and stained for E-cadherin (green). Confocal images were taken consecutively. Scale bar represents 50 µm. (3.00 MB TIF) [file pone.0004755.s006.tif]

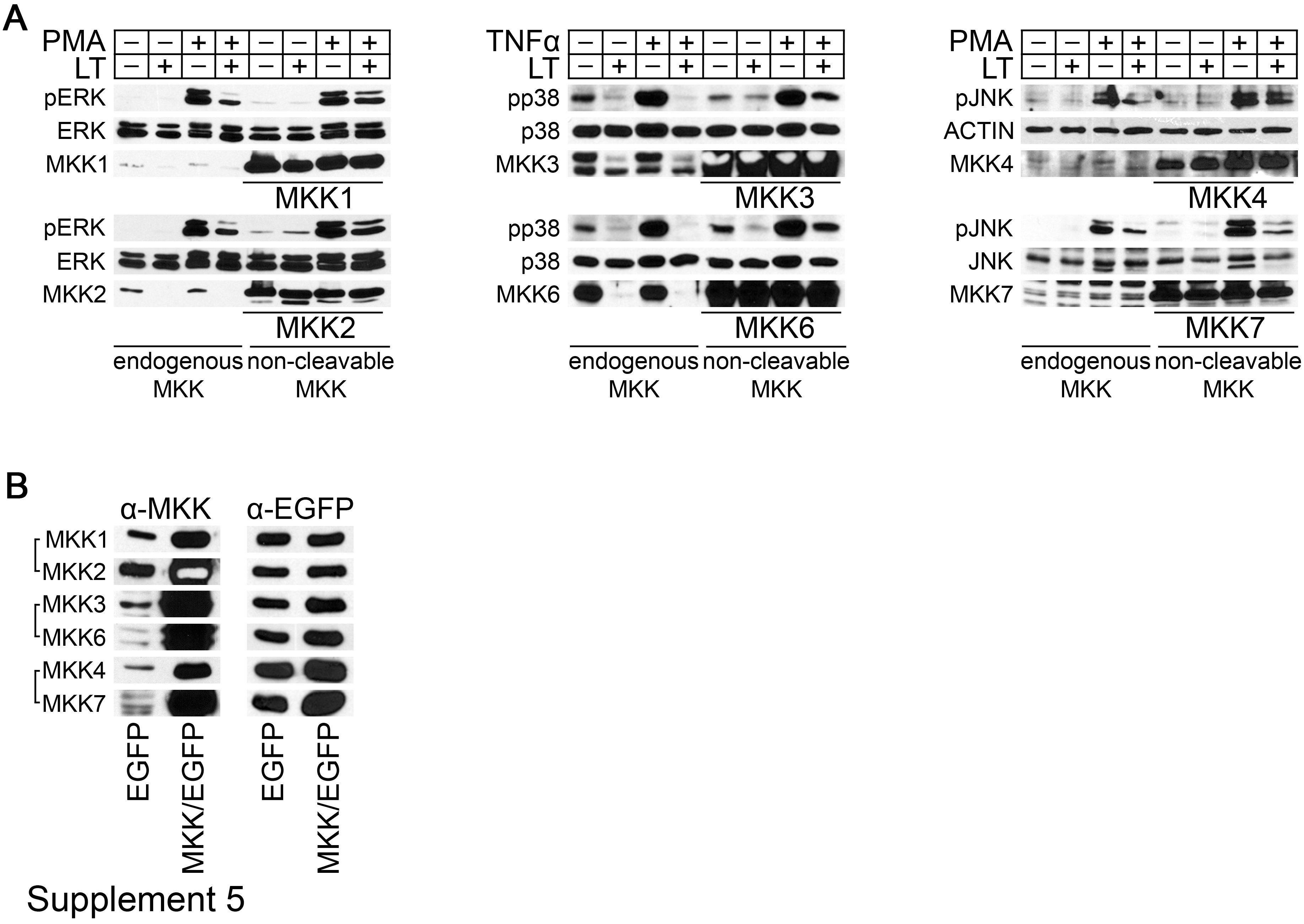

Supplement: Figure S5 — Validation of non-cleavable MKKs. (A) Non-cleavable MKK mutants or empty vector plasmid were transiently transfected into HeLa cells and expressed for 24 h followed by 24 h of LT treatment. Cells were then stimulated for 15 min with PMA (100 ng/ml) or TNFα (25 µg/ml) to activate MKK/MAPK pathways. Endogenous MKK expression; exogenous, non-cleavable MKK expression; LT-mediated MKK cleavage; phosphorylation of MAP kinases Erk, p38 or JNK; and total Erk, p38 and JNK expression are visualized by immunoblot as indicated. (B) Lentiviral expression of non-cleavable MKK pairs (MKK1/2, MKK3/6, MKK4/7) together with EGFP and of EGFP alone in SALE cells (see Methods). Lysates of the four SALE cell lines were probed by immunoblot with MKK specific and GFP antibodies. (1.45 MB TIF) [file pone.0004755.s007.tif]
